# P05-13 Associations between occupational physical activity and progression of carotid atherosclerosis among middle-aged women, are those with pre-existing cardiovascular disease more vulnerable?

**DOI:** 10.1093/eurpub/ckac095.080

**Published:** 2022-08-29

**Authors:** Mette Korshøj, Karen Allesøe, Ole Steen Mortensen, Volkert Siersma, Jussi Kauhanen

**Affiliations:** 1 Department of Occupational and Social Medicine, Holbæk Hospital, Holbæk, Denmark; 2 Center for Clinical Research and Prevention, Bispebjerg and Frederiksberg Hospital, Frederiksberg, Denmark; 3 Department of Public Health, Section of Social Medicine, University of Copenhagen, Copenhagen, Denmark; 4 Institute of Public Health and Clinical Nutrition, University of Eastern Finland, Kuopio, Finland

**Keywords:** Cardiovascular diseases, Cardiovascular mortality, Epidemiology, Population-based, Prospective study, Women, Work Environment, Prevention of cardiovascular disease, Occupational health and safety, Physical strain at work, Strenuousness of physical work

## Abstract

**Background:**

Workers with high occupational physical activity (OPA) experience higher risk of cardiovascular diseases (CVD) than sedentary workers. In an ageing workforce, greater proportions will be expected to have CVD, and pre-existing CVD are shown to increase vulnerability to exposure of high OPA. However, most of the epidemiological evidence is derived from male cohorts, excluding those with pre-existing CVD, and using clinically manifest disease outcomes that are prone to healthy worker selection bias. To address these limitations and to highlight whether pre-existing CVD indicate vulnerability, this study investigated the effects of OPA on pre-clinical asymptomatic progression of carotid artery intima-media thickness (IMT) among women.

**Methods:**

Women participants (N = 905) of the population-based Kuopio Ischemic Heart Disease Study (KIHD) with data on OPA and IMT were included. Linear mixed models, stratified by pre-existing CVD, estimated the association between OPA and the IMT progression from baseline (1998-2001) through 8-years of follow-up.

**Results:**

Non-stratified analysis showed the greatest 8-year IMT progressions by exposure to light standing or moderately heavy active work (both 0.13 mm). Stratified analysis on within group changes and total level of IMT showed exposure to light standing and moderately heavy active work to give the greatest 8-year IMT progressions, especially pronounced among normotensives and those with pre-existing stenosis or ischemic heart disease (IHD). Women with pre-existing CVD ended up with the greatest total IMT levels (sum of baseline and estimated 8-year IMT change), in spite of less 8-year IMT change than among women with out pre-existing CVD. This may be explained by their initial high IMT level, combined with a celling effect of the change.

**Conclusion:**

Exposure to light standing work and moderately heavy active work was associated with accellerated progression of IMT, especially pronounced among normotensives or workers with pre-existing stenosis or IHD. The majority of the reported 8-year IMT progressions are at a clinically relevant magnitude of 0.1 mm, which associates with an 11% increased risk of acute myocardial infarction.

